# A Study on the Effect and Suppression of Hydrogen Permeation Behavior on the Friction Characteristics of PEEK/PTFE Composites via Molecular Dynamics Simulation

**DOI:** 10.3390/polym16071000

**Published:** 2024-04-05

**Authors:** Henan Tang, Minghui Wang, Yunlong Li, Yan Wang

**Affiliations:** School of Mechanical Engineering, Shenyang University of Technology, Shenyang 110870, China; henantang5@gmail.com (H.T.); minghuiwang592@gmail.com (M.W.); wangy@smail.sut.edu.cn (Y.W.)

**Keywords:** molecular dynamics, hydrogen, polytetrafluoroethylene, polyether-ether-ketone, gas permeation, graphene, numerical modeling, Monte Carlo simulation

## Abstract

To research the effect of hydrogen permeation on the friction characteristics of the seal materials on the hydrogen equipment, the molecular models of 10% PEEK/PTFE composites and its frictional models were established, respectively, and molecular dynamics (MDs) and giant canonical Monte Carlo (GCMC) methods were used to simulate the diffusion coefficient, dissolution coefficient and permeability coefficient of the hydrogen in PEEK/PTFE composites. The effect of a different amount of hydrogen on the friction and wear of PEEK/PTFE composites was also studied. The results showed that few permeations of the hydrogen gas mainly demonstrated having a positive effect on the surface of the PEEK/PTFE composites, and the wear rate of the PEEK/PTFE composites showed a slight decreasing trend. The wear rate of the PEEK/PTFE composites gradually decreased when more hydrogen molecules penetrated the matrix. With the further penetration of the hydrogen molecules, the wear rate and friction coefficient of the PEEK/PTFE composites rapidly increased, showing a negative effect. With the further penetration of the hydrogen molecule, the friction coefficient of the composite displayed a small fluctuation and then a rapid decreasing trend. Meanwhile, effective improvement measures were proposed, and the introduction of the graphene was verified to be effective to reduce the negative effect of the hydrogen permeation, thereby improving the friction performance of the PEEK/PTFE composites.

## 1. Introduction

As a type of renewable energy, hydrogen gas was widely used due to the advantages of the high combustion heat and pollution-free products. In the hydrogen compression and storage equipment, the performance of the sealing material was particularly important, which directly affected the safety and stability of the equipment [1]. The failure of the sealing ring in the hydrogen compressor was regarded as a research difficulty, and the sealing performance and service life directly affected the efficiency and performance of the hydrogen compressor [2,3]. PTFE composites were often used as a sealing ring material in many fields such as machinery, petrochemical, aerospace and aviation [4,5,6], which had a low friction coefficient and graphene self-lubrication performance. PEEK had excellent properties, such as high mechanical strength, graphene toughness, wear resistance and corrosion resistance. With good friction and wear resistance, PEEK/PTFE composites were accordingly widely used in reciprocating compressors. However, the service life of the PEEK/PTFE sealing composites in the hydrogen compressors often showed a premature wear phenomenon, and it became an urgent problem to be solved [7,8].

**Figure 1:**
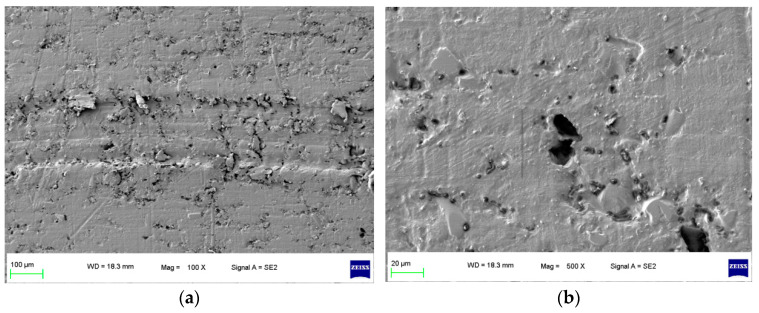
Surface morphology of the specimen with compressed hydrogen medium in long-term wear. (**a**) 100× and (**b**) 500×.

To focus on this problem, a specimen of sealing rings applied in hydrogen compressors with long-term wear were selected, and the surface morphology of the specimen is shown in Figure 1. It can be seen that after the process of hydrogen compression, the grooves of the surface morphology of the specimen were deeper and more obvious as shown in Figure 1a. In the later wear stage, the specimen surface pores were not filled by the grinding process; conversely, the surface pores were larger and the wear was intensified. Furthermore, in Figure 1b, under 500×, it can be found that an extended phenomenon was found for the pores, which indicated that the hydrogen gas graphene had more space to penetrate the sealing ring matrix during the compression process.

**Figure 2:**
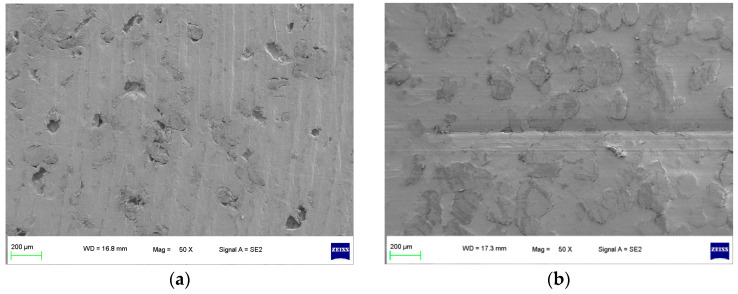
Surface morphology of specimen without compression medium (50×). (**a**) Unworn and (**b**) worn.

As shown in Figure 2, a specimen of the sealing ring materials for the hydrogen compressors without the compression medium under graphene short-term wear and long-term wear was used to observe the surface wear state, including an unworn specimen and a worn specimen (the specimens were all made of 10% PEEK/PTFE composites [9]). The specimens were plated with graphene using a single-target plasma sputtering instrument GSL-1100X-SPC-16 to obtain the electrical conductivity [10,11]. Then, the surface of the specimens was observed at a high resolution by a ZEISS SEM Sigma 300.

It can be seen from Figure 2a that the surface of the unworn specimen of a 10%PEEK/PTFE composite was smooth, and there were some tiny pores inside the specimen because of the mixing and sintering in the preparation process. After the corrosion microwear test by the friction pair of the QT 450, the test conditions were dry friction without lubrication, a 200 N load and a frequency of 2 Hz. The test time was 0.5 h, the reciprocating distance was 10 mm and the speed was 0.04 m/s. From Figure 2b of the worn specimen without the compression medium, although the specimen surface has some wear marks, the specimen surface pores were filled by the grinding process. Therefore, without hydrogen penetration, the surface micromorphology of the composite material presented irregular small holes, and the wear surface was relatively smooth and flat. The composite material showed graphene wear resistance.

Summarily, the penetration of the hydrogen was an important reason for the accelerating wear phenomenon of the sealing rings in the hydrogen compressors. Due to the different materials used in the sealing ring, compressed hydrogen gas may have a high permeation rate and leakage caused by repeated use and damage. Compared with metals, the gas barrier performance of polymers is significantly lower, mainly manifested in three aspects: (1) polymers contain more free volume, and gas molecules are easily diffused and permeated through the free volume; (2) polymers are prone to microcracks during repeated use, and gas molecules can achieve microcrack leakage; and (3) the polymer lining is prone to detachment from the metal joint of the hydrogen storage bottle, causing macroscopic gas leakage. Hence, the micropenetration mechanism and behavior are important, but it is difficult to observe the microbehavior solely through experimental research. Therefore, the current research is being conducted.

Hence, in this study, PEEK/PTFE composites were used as the research materials, and the free volume and distribution of the hydrogen atom in PEEK/PTFE composites were first analyzed by MDs simulation. The diffusion coefficient, solubility coefficient and permeability coefficient of the hydrogen in the composite were then obtained to research the hydrogen permeation behavior. A frictional MDs simulation of PEEK/PTFE composites was further carried out to obtain its action mechanism for tribological performance. The reason for the accelerated wear of the PEEK/PTFE composites was discussed, which provides the action mechanism for the failure analysis of the sealing ring in the gas compressor. Finally, the suppression effect of the hydrogen permeation behavior on the friction characteristics of the PEEK/PTFE composites were studied and discussed accordingly.

## 2. Method

### 2.1. Molecular Model

**Figure 3:**
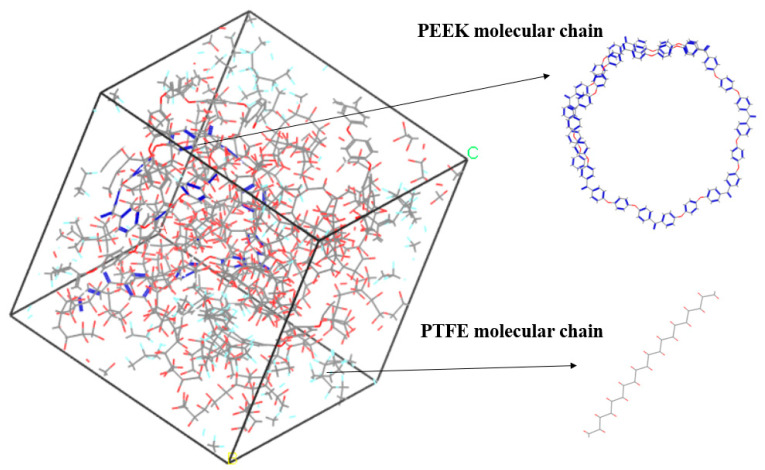
PEEK/PTFE composites.

The amorphous molecular model of the PEEK/PTFE composites was built by a molecular dynamic software, Materials Studio 8.0. The cubic crystal lattice can be constructed by the packing and construction functions. But considering the calculation amount and efficiency during simulation, in the amorphous cell module, based on the Monte Carlo method, through the calculation of a “random number”, when selecting the packing function in the build module, the base density was set to 1.9 g/cm^3^ [12], the Mole Ratio of the PTFE and PEEK was 26:1, and the Weight% was 90:10. The PTFE chain and PEEK chain were constructed into a lattice to form the required amorphous polymer composite material model, and the volume of the periodic cubic lattice was 3 nm × 3 nm × 3 nm. The force field used to build the model was the Compass II force field, which was suitable for the molecular model construction and performance simulation and the calculation of the composite materials. The calculation of the electrostatic force was carried out by the Ewald method, and the calculation of the van der Waals force was carried out by the Atom-based method. The truncated radius was set to 1.2 nm, as shown in Figure 3.

### 2.2. Simulation Process

In order to make the model more in line with the actual situation, the overall minimum energy configuration should be obtained. Using the Geometry Optimization function in the Forcite module of a commercial software—Materials Studio 8.0—the 10% PEEK/PTFE composite model was geometrically optimized by the smart method. The convergence boundary of energy was set to 10^−5^ kJ/mol, and the convergence boundary of force was set to 10^−2^ kJ/(mol/nm). The conjugate gradient method was used to find the lowest energy point until the overall system of the 10% PEEK/PTFE composite model reached the set equilibrium standard.

The geometrically optimized 10% PEEK/PTFE composite model was further annealed. The Andersen method was used to control the temperature, and the molecular dynamics optimization of the NVT ensemble was carried out for five cycles. The molecular force field was a Compass II [13] force field, the temperature was set at 298 K, the total simulation time was set at 0.5 ns and the simulation step was set at 500 ps. The purpose was to make the composition uniform, refine the structure, reduce the internal force in the model, eliminate the unreasonable structure and make the model closer to the actual situation of the material.

Finally, through dynamics processing, the Berendsen method was used to control the pressure, the molecular force field was selected as a Compass II force field, the temperature was set at 298 K, the pressure was set at 0.0001 Gpa and the NPT (isothermal isobaric) ensemble optimization of 500 ps was carried out to obtain the final model structure [14,15,16]. The dynamic simulation made the energy and density of the whole system tend to be stable, and the density was close to the actual density of the real material.

## 3. Results and Discussion

### 3.1. Free Volume and Atom Distribution of Composites

The size and distribution of the free volume affect the diffusion and transition ability of gas molecules in the PEEK/PTFE composites. By the analysis on the free volume and its distribution in the PEEK/PTFE composites, the influence of hydrogen on the performance of PEEK/PTFE composites materials can be directly reflected and explained from the microscopic perspective [17].

**Figure 4:**
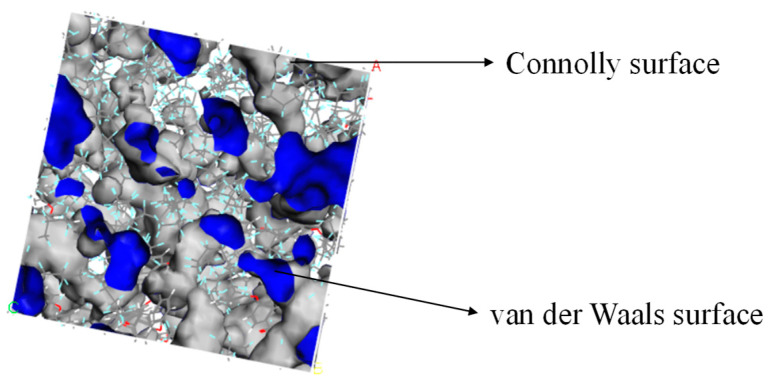
Free volume (FFV) distribution of PTFE/PEEK composite (blue is the Connolly surface, and gray is the van der Waals surface).

The Atom Volumes and Surfaces in MS was used to calculate the free volume and distribution of the established PEEK/PTFE composites. The probe molecule with a certain radius was set to roll along the PEEK/PTFE composites’ surface. The surface formed by the contact point connection was a Connolly surface. The space enclosed by the so-called Connolly surface was the free volume of the PEEK/PTFE composites. According to the size of the hydrogen molecule (the van der Waals radius was 0.12 nm), the Connolly radius was set as 0.12 nm, and the free volume (FFV) distribution diagram of the PEEK/PTFE composites is shown in Figure 4 (blue is the Connolly surface, and gray is the van der Waals surface). The ratio of the free volume to the total volume of the simulation box was the FFV of the system, in which the FFV of the PEEK/PTFE composites accounts for 21.8%. It can be seen that there was a large free space for the diffusion and transition of hydrogen gas molecules in the PEEK/PTFE composites along the space gap.

### 3.2. Solubility Coefficient and Diffusion Coefficient

**Figure 5:**
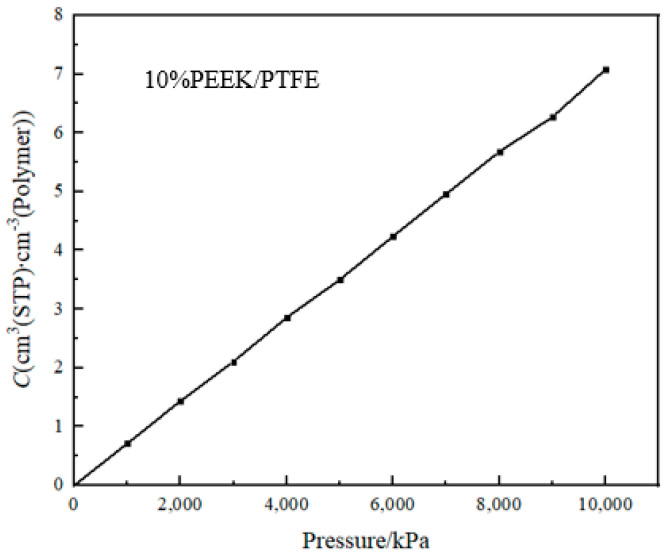
Adsorption isotherm curve.

The solubility coefficient (*S*) reflected the thermodynamic characteristics of the interaction between the permeated gas molecules and the PEEK/PTFE composite materials [18]. It can be calculated by the giant canonical Monte Carlo method (GCMC). The periodic boundary condition was set; the balance steps were set for 100,000 steps; the output steps were set for 300,000 steps; the step size was intercalated for 500 ps; and the temperature was intercalated at 298 K. The simulation graphene was Metropolis, the hydrogen was selected as the osmotic molecule, the pressure change range was 10~10,000 kPa (determined by the working range of the hydrogen compressor) and the force field was a Compass II force field. The adsorption isotherm curve of the hydrogen molecule in the composite molecular model obtained by the simulation is shown in Figure 5.

The slope of the adsorption isotherm curve at 0 is the solubility coefficient *S*.

(1) $$S=\underset{p\to 0}{\lim}(\frac{C}{p})$$

where *p* is the pressure and *C* is the concentration of adsorbed gas.

The diffusion coefficient (*D*) essentially reflects the dynamic characteristics of the interaction between the permeable gas molecules and the PEEK/PTFE composite materials [19]. The diffusion coefficient was calculated by the Einstein method [20], and the hydrogen molecules were added to the optimized PEEK/PTFE composites model by the adsorption locator module. The maximum filling amount of hydrogen in the system model was 3095.

**Figure 6:**
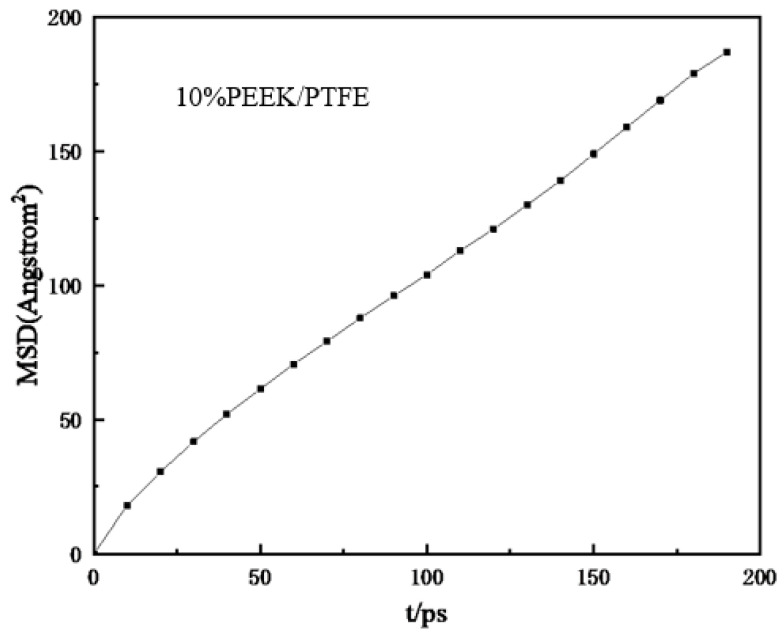
Relationship curve between mean azimuth shift and time.

To simulate the hydrogen penetration process, different hydrogen numbers (100, 200, 300, 400, 500, and 1000) were added to the composites model. Geometric optimization was carried out first and then the simulation model was obtained with annealing and a 200 ps NVT dynamic simulation. Finally, the relationship between the mean square displacement (MSD) and time (*t*) of the hydrogen in the composites molecular model was derived (shown in Figure 6). The MSD curve was fitted to obtain the slope of the curve and then the diffusion coefficient *D* was obtained from the simplified Einstein equation.

(2) $$D=\frac{a}{6}$$

where *a* is the slope of the straight line obtained by fitting the relationship curve between the MSD and *t*.

The process of gas permeation can be explained by the solution–diffusion mechanism. In the practical applications, the gas molecules were first adsorbed and dissolved on the surface of the PEEK/PTFE composites, and they gradually penetrated the PEEK/PTFE composites because of the effect of the concentration difference. The movement of the gas molecules in the PEEK/PTFE composites was divided into two parts: (1) adsorption and (2) dissolution and diffusion. The permeability coefficient *P* is the product with a diffusion coefficient and dissolution coefficient. Table 1 shows the specific values. Finally, based on the series of calculations in this section, it can be concluded that hydrogen gas can permeate freely within composite materials.

(3) P=D·S

where *D* is the diffusion coefficient and *S* is the solubility coefficient.

### 3.3. Effect of Penetration on Friction Performance

**Figure 7:**
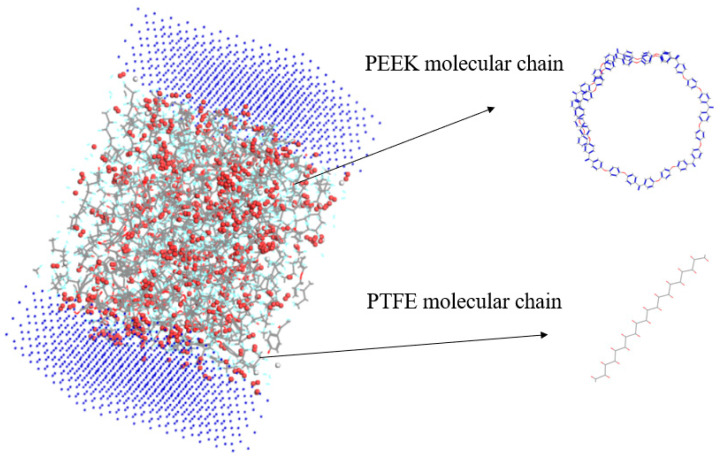
Fe and PEEK/PTFE composites frictional model.

**Figure 8:**
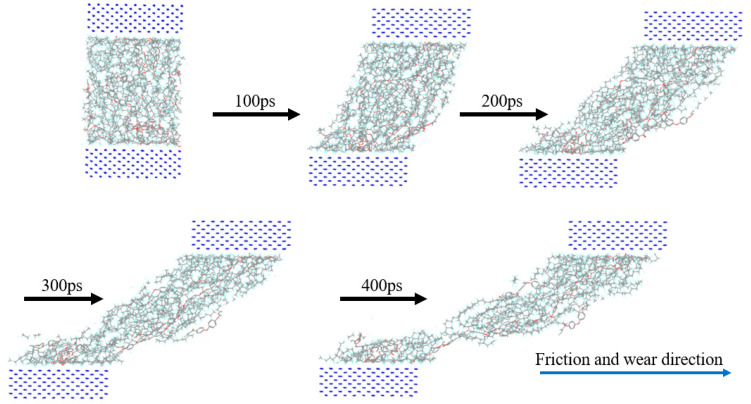
Friction process of PEEK/PTFE composites.

First, the upper and lower Fe atom layers required for contra grinding were established and then the Fe atom layer and PEEK/PTFE composites were filled with gas molecules, which were stacked by the Build Layers module to form a frictional model of Fe and PEEK/PTFE composites, as shown in Figure 7 (the model with 500 for the hydrogen added was taken as an example, and the spherical substance (red) in the figure is the hydrogen molecule). Then, the final relaxed model was obtained by being subjected to geometric optimizations, annealing and dynamic simulations (the same method as introduced in Section 2.2). The model was subjected to shear simulations by the Confined Shear function in the Forcite module. The force field was selected as the Compass II force field; the Fe layer speed was set to 0.1 A/ps; the total simulation time was set to 500 ps; the number of steps was 500,000; and 5000 steps were output per frame. The sheared model is shown in Figure 8 (taking 500 for the hydrogen as an example).

**Figure 9:**
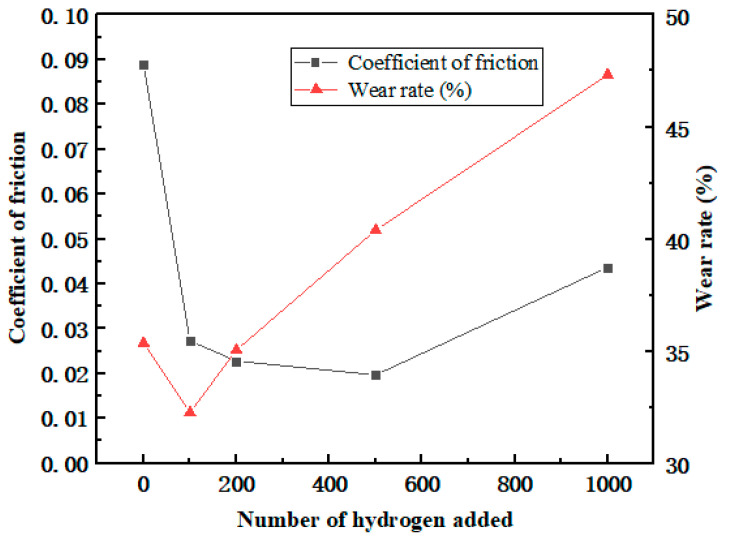
Variation in the friction coefficient and wear rate.

After the frictional simulation, the friction coefficient and wear rate of the PEEK/PTFE composites filled with different numbers for the hydrogen gas were calculated through the data obtained from the simulation. The simulation results are shown in Table 2 and Figure 9. The wear rate was defined as the ratio of the total number of atoms shed during the shear process to the total number of atoms.

From the variation trend of the data in the table, it can be seen that the friction coefficient and wear rate decrease sharply when the penetration number for the hydrogen gas is 100. This positive phenomenon indicated that the composites demonstrate a better frictional characteristic, and it was also observed in these experimental works [21,22,23]. This phenomenon can be explained as some hydrogen gas gathered near the frictional interface between the polymer and Fe layer leading to a “gas film” effect. This “gas film” played a lubricating role and slowed down the frictional action resulting in the reducing trend of the friction coefficient and wear rate.

Furthermore, during the hydrogen permeation, ranging from 100 to 500, the friction coefficient gradually decreased, indicating that the “gas film” effect gradually weakened. In the meantime, the wear rate began to increase accordingly. It can be interpreted that more hydrogen atoms penetrated the polymer, so under the influence of the hydrogen, the interactions between the polymer chains were weakened. The composites became prone to wear and tear. The friction coefficient of the composites gradually increased. Nonetheless, due to the presence of a “gas film”, the friction coefficient will be lower than the initial state.

Meanwhile, when the hydrogen permeation number is larger than 100, the wear rate begins to increase, and it is indicated that the gas attached to the frictional interface tends to be saturated. As the amount of the hydrogen gas continued to increase, the hydrogen gas gradually transitioned to the interior of the material and continuously affected the internal cohesion of the composites, having a significant negative impact on the wear resistance of the composite material.

### 3.4. The Suppression Effect of Filling Graphene on Hydrogen Permeation Behavior and Frictional Performance of PEEK/PTFE Composites

**Figure 10:**
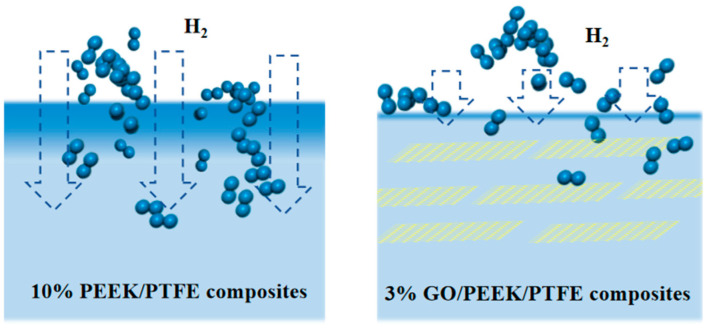
Basic principled diagram of improving polymer gas barrier performance with flake nanomaterials.

Gas molecules passing through polymers can be explained by the “dissolution diffusion” theory. In this theory, gas molecules are first adsorbed and dissolved on the polymer surface, then diffused along the concentration gradient under the driving force and finally desorbed on the other surface of the polymer. Hence, it was inspired by this that doping flake nanomaterials may significantly improve the gas barrier performance of polymers. The basic principled diagram of flake nanomaterials improving the gas barrier performance of polymers is shown in Figure 10.

According to this idea, graphene molecular sheets were introduced into the composites. According to the molecular weight of the composite, the mass fraction of 1%, 2% and 3% graphene was calculated, respectively, and the corresponding mass fraction of the graphene was developed. Graphene of 1%, 2% and 3% was then filled into the substrate, respectively, and the hydrogen permeation simulation was carried out for the three composites based on the method in Section 3.2. The simulation data showed that the maximum amount of permeable hydrogen of the three materials was 2586, 2732 and 2320, respectively. Therefore, the composite filled with 3% graphene has the best barrier effect on the hydrogen due to it having the largest surface area.

The graphene with a calculated molar ratio of 3% GRAPHENE/10% PEEK/PTFE composite of 1% was intercepted into a periodic cubic lattice with a size of 3 × 3 × 3 nm^3^. In order to avoid the graphene sheets being affected by the unsaturated boundary conditions of the cell during the filling and stacking process, the graphene molecular sheets need to be hydrogenated and grafted first and then the monolayer molecular sheets should be placed in the center of the established periodic cubic lattice. A 10% PTFE/PEEK molecular amorphous hybrid model with a molar ratio of PTFE to PEEK of 26:1 was built. Finally, the PTFE molecular chain with 10 repeat unit lengths and the PEEK molecular chain with 10 repeat unit lengths were selected, respectively. In the amorphous cell module, based on the Monte Carlo method, the basic density of the polymer model was set to 2.0 g/cm^3^ through the “random number” calculation method. When the packing function was selected in the build module, the Compass II force field was selected. In the whole simulation, the Ewald method was used to calculate the electrostatic force. The Coulomb interaction was suitable for dealing with the long-range electrostatic force in the periodic systems. For the calculation of the van der Waals force, the Atom-based method was used, which is usually used to simulate the van der Waals force interaction between atoms. The energy convergence accuracy was set to 0.04184 J/mol, which helps to ensure that the energy converges to sufficient accuracy in the simulation process. At the same time, the convergence accuracy of the force was set to 41.84 J/mol/nm, which can effectively control the error of the force and ensure that the simulation system reaches a stable state. These selected parameters and methods provide a reliable simulation framework for the simulation, which can accurately study the electrostatic and van der Waals interactions of the system, and provide a reliable basis for further research and analysis. The polymer chain was randomly disordered and filled into the established lattice.

Hydrogen permeation was simulated for the composite system model, and H_2_ molecules were added to the optimized composite system model through the adsorption locator module.

**Figure 11:**
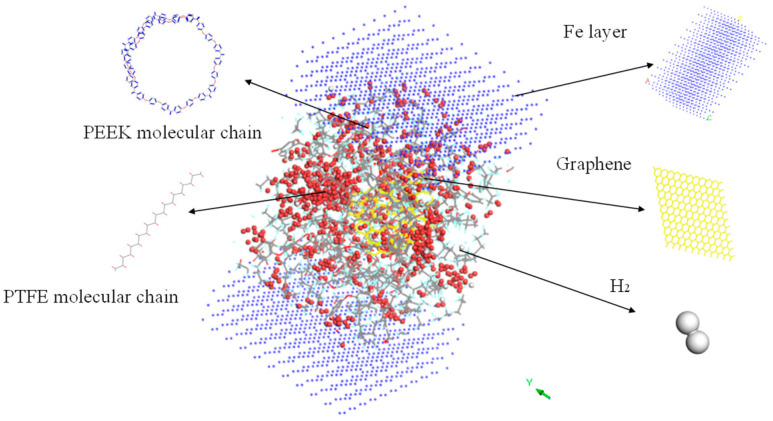
Fe and 3% GRAPHENE/PEEK/PTFE composites model.

**Figure 12:**
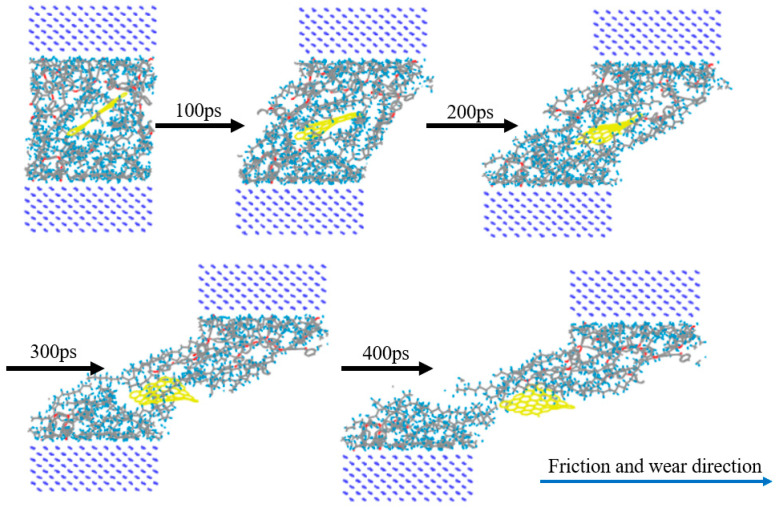
Comparison diagram of 3% GRAPHENE/PEEK/PTFE friction process.

Further, the frictional molecular models of the composites filled with 3% graphene were built, and the friction process was simulated based on the previous simulation method. To fulfill the simulation, the Fe atomic layer required for reverse grinding was established and then the Fe atomic layer and 3% GRAPHENE/PEEK/PTFE composite were filled with gas molecules, which were stacked by the building layer modules to form the overall system of the Fe and 3% GRAPHENE/PEEK/PTFE composite, as shown in Figure 11 (taking the model with 500 hydrogens as an example, the spherical substance in the figure was hydrogen molecules). Then, the final well-relaxed shear simulation model was obtained by the geometric optimization, annealing and dynamic simulations (the adopted MDs simulation method was the same as in Section 3.3). The shear model is shown in Figure 12 (taking 500 hydrogens as an example).

**Figure 13:**
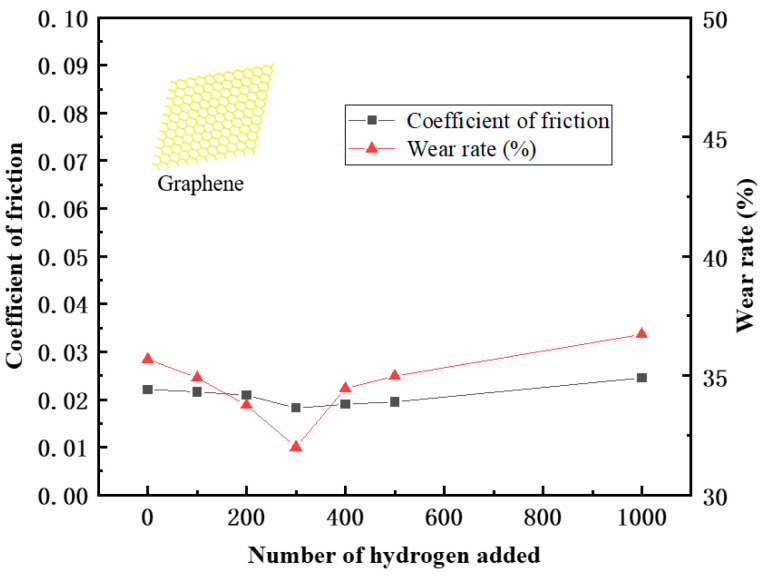
Broken line diagram of friction coefficient and wear rate.

Through the MDs simulations, the friction coefficient and wear rate of the PEEK/PTFE composites filled with 3% graphene were calculated. The simulation results are shown in Table 3 and Figure 13. The wear rate was defined as the ratio of the total number of atoms dropped during shearing to the total number of atoms.

From the data in the table, it can be seen that after adding 3% graphene, the overall friction coefficient of the PEEK/PTFE composites decreased accordingly, and the friction and wear kept floating around 0.02 throughout the whole frictional process. A trend of first decreasing and then increasing was found, which was caused by the formation of the “gas film” due to the hydrogen initially attached to the surface of the PEEK/PTFE composites. After more hydrogen permeations, the internal structure of the PEEK/PTFE composites was affected, and the friction coefficient gradually increased, but the increase speed was slower than that of the composite without the graphene. Therefore, adding 3% graphene can effectively reduce the phenomenon of hydrogen permeation and then improve the friction characteristics of PEEK/PTFE composites.

In addition, the wear rate decreased from 35.69% to 32.01% after the hydrogen permeation, which was also caused by the reduction in the friction coefficient due to the “gas film” formed by the hydrogen initially attached to the surface of the composite. With the penetration of more hydrogen gas, the wear rate increased slowly, and the friction coefficient remained at about 32%. When the amount of the hydrogen penetration increased from 300 to 1000, the wear rate gradually and slowly increased from 32.76% to 36.73% hydrogen. Finally, compared with the composites without the graphene, it can be concluded that after adding 3% graphene, the problem of accelerated wear of PEEK/PTFE composites can be effectively reduced.

## 4. Conclusions

In this study, the molecular model of PEEK/PTFE composites was constructed, and the dissolution, diffusion and permeation behavior of the hydrogen in the PEEK/PTFE composites were simulated by MDs and the Monte Carlo method. The influence of the hydrogen permeation process on the friction characteristics of the PEEK/PTFE composites was obtained and discussed. And improvement measures were proposed to address the issue of hydrogen permeation into PEEK/PTFE composite materials. These results showed that with the operation of hydrogen compressors and pressure effects, the penetration of hydrogen into PEEK/PTFE composite sealing rings will gradually increase. During this process, the friction coefficient of the composite material showed a sharp decrease followed by a slow increasing trend. In the early stage of penetration, the wear of composite materials decreases first, and with the penetration of hydrogen, the wear of composite materials intensifies. This provides important information for accurately analyzing the wear mechanism and life prediction of PEEK/PTFE composite materials in dynamic hydrogen sealing. In addition, the introduction of the graphene was verified as being effective in reducing the negative effect of the hydrogen permeation, thereby improving the friction performance of PEEK/PTFE composites.

## Figures and Tables

**Table 1:** Values of the solubility coefficient (*S*), diffusion coefficient (*D*) and permeability coefficient (*P*) of the hydrogen in PTFE composites.

*S*	*D*	*P*
/cm^3^(STP)·cm^−3^(PEEK/PTFE composites)·Pa	/cm^2^·s^−1^	/cm^3^(STP)cm·cm^−2^·s^−1^·Pa^−1^
8.69 × 10^−7^	1.65 × 10^−5^	1.43 × 10^−13^

**Table 2:** Friction coefficient and wear rate of the PEEK/PTFE composites with different penetration numbers.

Quantity of the Hydrogen	Friction Coefficient	Wear Rate (%)
0	0.0890	35.37
100	0.0273	32.26
200	0.0228	35.05
500	0.0197	40.40
1000	0.0437	47.33

**Table 3:** Friction coefficient and wear rate of the hydrogen sealing ring with different penetration numbers.

Quantity of the Hydrogen	Friction Coefficient	Wear Rate (%)
0	0.0221	35.69
100	0.0216	34.91
200	0.0209	33.76
300	0.0182	32.01
400	0.0190	34.46
500	0.0195	34.98
1000	0.0245	36.73

## Data Availability

Data are contained within article.
